# Sarcolipin-SERCA uncoupling as a plausible heat source for sustaining fever

**DOI:** 10.3389/fphys.2026.1865484

**Published:** 2026-06-24

**Authors:** A. Humaid, A. R. Tupling

**Affiliations:** Department of Kinesiology & Health Sciences, University of Waterloo, Waterloo, ON, Canada

**Keywords:** Ca2+ cycling, febrile thermogenesis, non-shivering thermogenesis, skeletal muscle, thermoregulation

## Abstract

Fever is an evolutionarily conserved host defence that elevates defended core temperature and metabolic rate, yet the dominant thermogenic tissue sustaining febrile heat production in adult mammals remains unresolved. Brown adipose tissue and shivering thermogenesis are emphasized in classical models, but adult BAT is quantitatively limited, and shivering is often intermittent and behaviourally costly. Because skeletal muscle is high-mass and widely distributed, skeletal muscle non-shivering thermogenesis is positioned as a viable contributor to febrile hypermetabolism, particularly during the maintenance phase. Febrile responses persist in UCP1-deficient models, while UCP3 disruption blunts LPS-induced hyperthermia, consistent with a muscle-linked contribution to febrile thermogenesis. Given that UCP3 is generally viewed as a metabolic-support uncoupler rather than a dedicated adaptive heat generator, sarcolipin-mediated SERCA uncoupling emerges as a plausible, scalable alternative. Inflammatory challenges are associated with increased muscle SLN expression without parallel SERCA expansion. These observations motivate integrated calorimetry, electromyography, and tissue-specific perturbation approaches to quantify skeletal muscle heat production during fever and distinguish putative SERCA uncoupling from shivering across translational models.

## 1 Introduction

Fever is an evolutionarily conserved host defence that elevates defended core temperature and whole-body metabolic rate, yet the principal thermogenic tissue supporting adult febrile heat production remains uncertain (Kluger et al., 1975; Evans et al., 2015; Walter et al., 2016). In mammals, fever commonly increases core temperature by about 1 to 4 °C, and this shift carries a substantial energetic cost on the order of 10 to 12.5% higher metabolic rate per 1 °C rise, with concordant increases in oxygen consumption (Du Bois, 1921; Evans et al., 2015). Although immune-to-brain signalling is well defined, it remains less clear how heat production is partitioned across peripheral thermoeffectors once the higher defended temperature is established (Engblom et al., 2003; Lazarus et al., 2007; Blomqvist and Engblom, 2018). Classical accounts emphasize brown adipose tissue (BAT) and shivering thermogenesis (ST) as the dominant effectors, but adult BAT is quantitatively limited and heterogeneous, and shivering is intermittent and behaviourally costly (Lenhardt et al., 1999; Cannon and Nedergaard, 2004; Cypess et al., 2009; Saito et al., 2009; van Marken Lichtenbelt et al., 2009; Virtanen et al., 2009; Haman and Blondin, 2017). This uncertainty supports the need for an evaluation of whether skeletal muscle contributes meaningfully to non-shivering thermogenesis (NST) during fever and thereby accounts for a substantial fraction of febrile energy expenditure in adult mammals.

**Figure 1:**
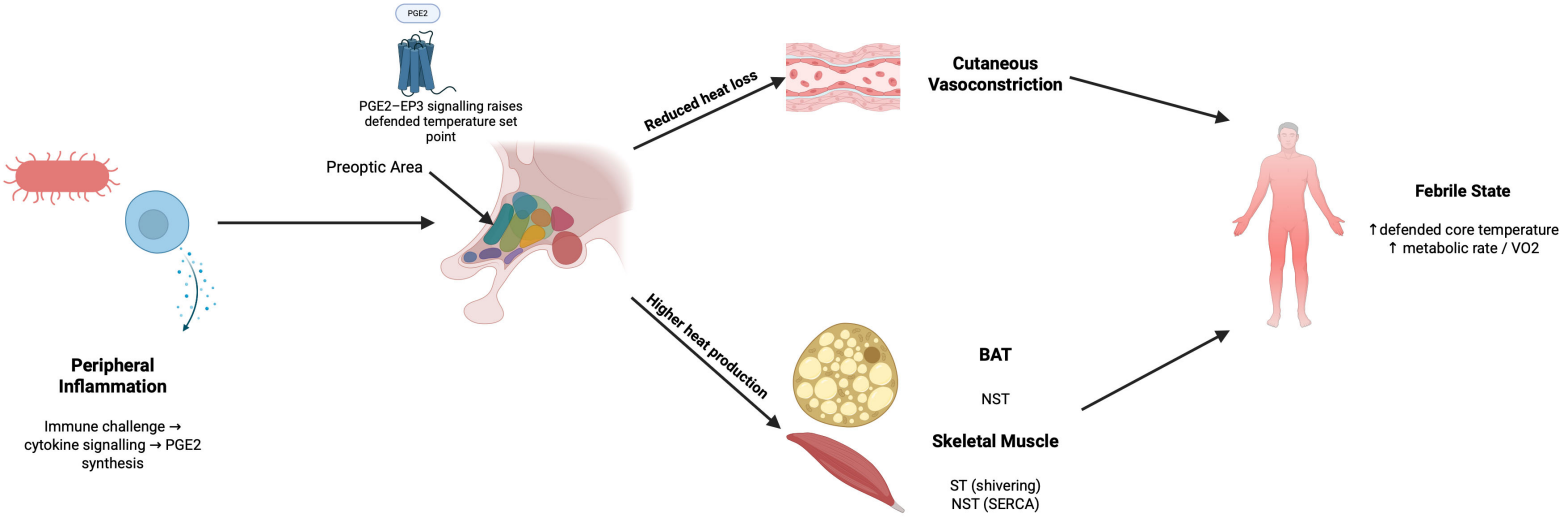
Schematic overview of fever generation following inflammatory challenge. Peripheral inflammatory signalling promotes PGE_2_-dependent action in the preoptic area, raising the defended temperature set point. This centrally commanded state is sustained by coordinated recruitment of thermoeffector pathways that reduce heat loss, including cutaneous vasoconstriction, and increase heat production through brown adipose tissue and skeletal muscle, resulting in elevated core temperature and metabolic rate. Made using BioRender.

Once inflammatory signalling raises the defended temperature set point through PGE_2_ action in the preoptic area, the organism enters a centrally commanded state that must be sustained by higher heat conservation and higher heat production (Engblom et al., 2003; Lazarus et al., 2007; Blomqvist and Engblom, 2018; **Figure 1**). For a typical adult, this translates into an added energetic requirement of a few hundred kilocalories per day and often coincides with infection-associated anorexia (Manthous et al., 1995; Exton, 1997; Dantzer et al., 2008; Wright and Auwaerter, 2020; Pontzer et al., 2021). The key unresolved question is how this heat production and energetic load are partitioned across tissues during the maintenance phase of fever, when the higher defended temperature is sustained after the initial rise and before resolution (Cannon and Nedergaard, 2004; Cypess et al., 2009; Saito et al., 2009; van Marken Lichtenbelt et al., 2009; Virtanen et al., 2009; Ouellet et al., 2012; Haman and Blondin, 2017; Morrison and Nakamura, 2019).

**Figure 2:**
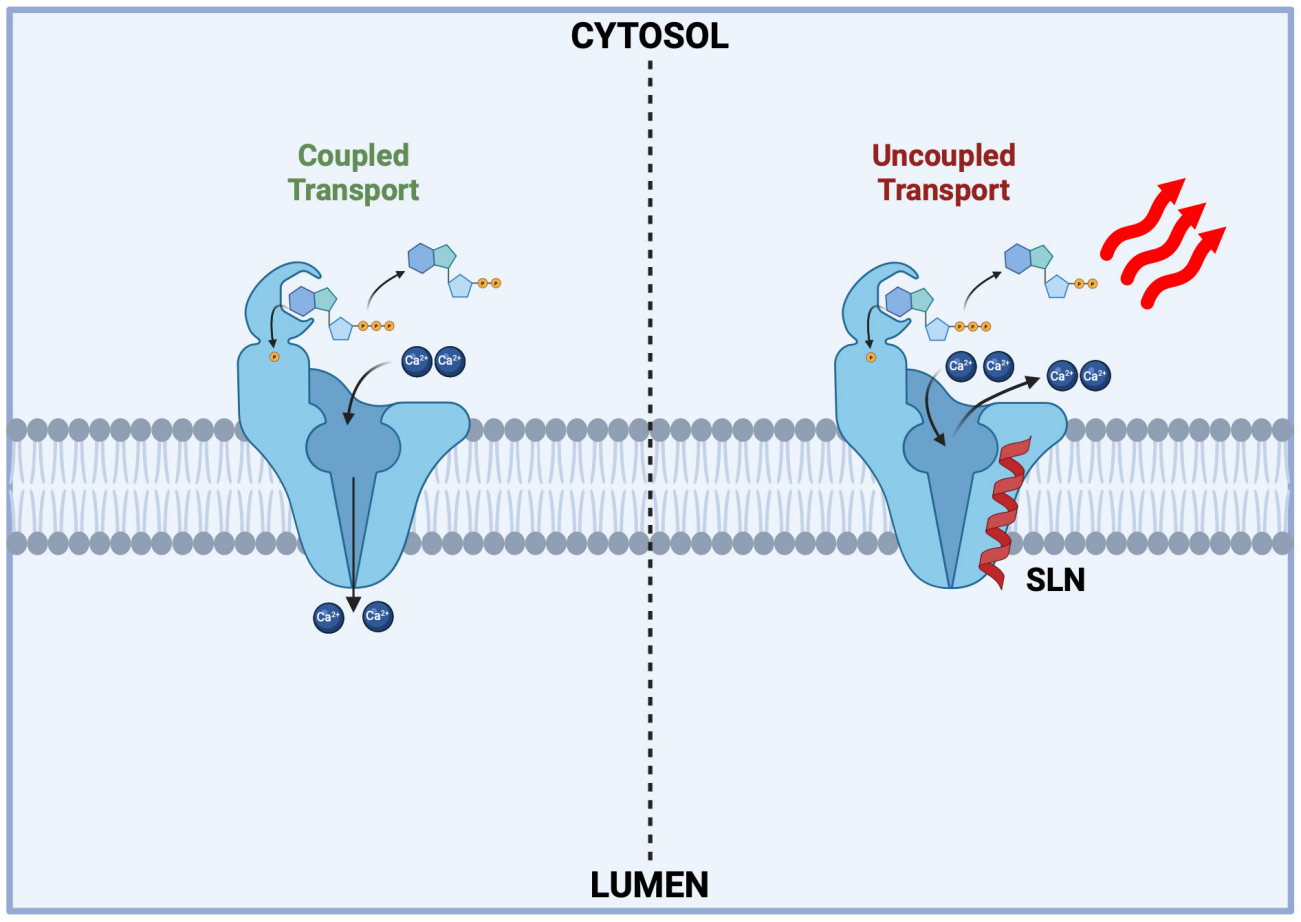
Conceptual model of coupled versus SLN-associated uncoupled SERCA Ca^2+^ cycling. In the coupled state (left), ATP hydrolysis drives SERCA to transport Ca^2+^ into the sarcoplasmic reticulum lumen. In the uncoupled state (right), SLN promotes Ca^2+^ slippage/futile cycling, lowering the Ca^2+^/ATP coupling ratio as ATP hydrolysis continues and a larger fraction of ATP-derived energy is dissipated as heat. Made using BioRender.

In this context, skeletal muscle NST can be treated as “extra” heat production that is ATP-driven and generated in muscle but not expressed as overt mechanical work (Stewart et al., 2010). The key distinction is that ATP turnover rises in resting fibres, with little sustained cross-bridge cycling and minimal evidence of ongoing motor-unit recruitment compared with ST (Haman and Blondin, 2017). A contraction-independent muscle heat source is therefore plausible on simple scaling grounds, because skeletal muscle comprises roughly 30–40% of adult body mass and small shifts in resting ATP demand can translate into meaningful whole-body heat production (Zurlo et al., 1990; Smith et al., 2013; Periasamy et al., 2017). Mechanistically, current models converge on sarcoplasmic reticulum Ca^2+^ cycling via SERCA as the leading effector class for muscle NST (Periasamy et al., 2017; Gamu et al., 2020). Ca^2+^ leak and reuptake continue in the absence of contraction, and SERCA ATP hydrolysis can represent a substantial fraction of daily energy turnover, creating leverage for thermogenesis without force production (Smith et al., 2013; Gamu et al., 2020). If SERCA coupling efficiency is reduced, a larger fraction of ATP free energy is dissipated as heat per unit Ca^2+^ transported (Gamu et al., 2020; **Figure 2**). Sarcolipin (SLN) is central to this concept, because it can bias SERCA toward low-efficiency, heat-producing cycles while preserving Ca^2+^ homeostasis, thereby increasing heat release without requiring overt contraction (Smith et al., 2002; Bal et al., 2018). However, direct evidence that this mechanism is a dominant source of febrile heat production remains lacking.

**Figure 3:**
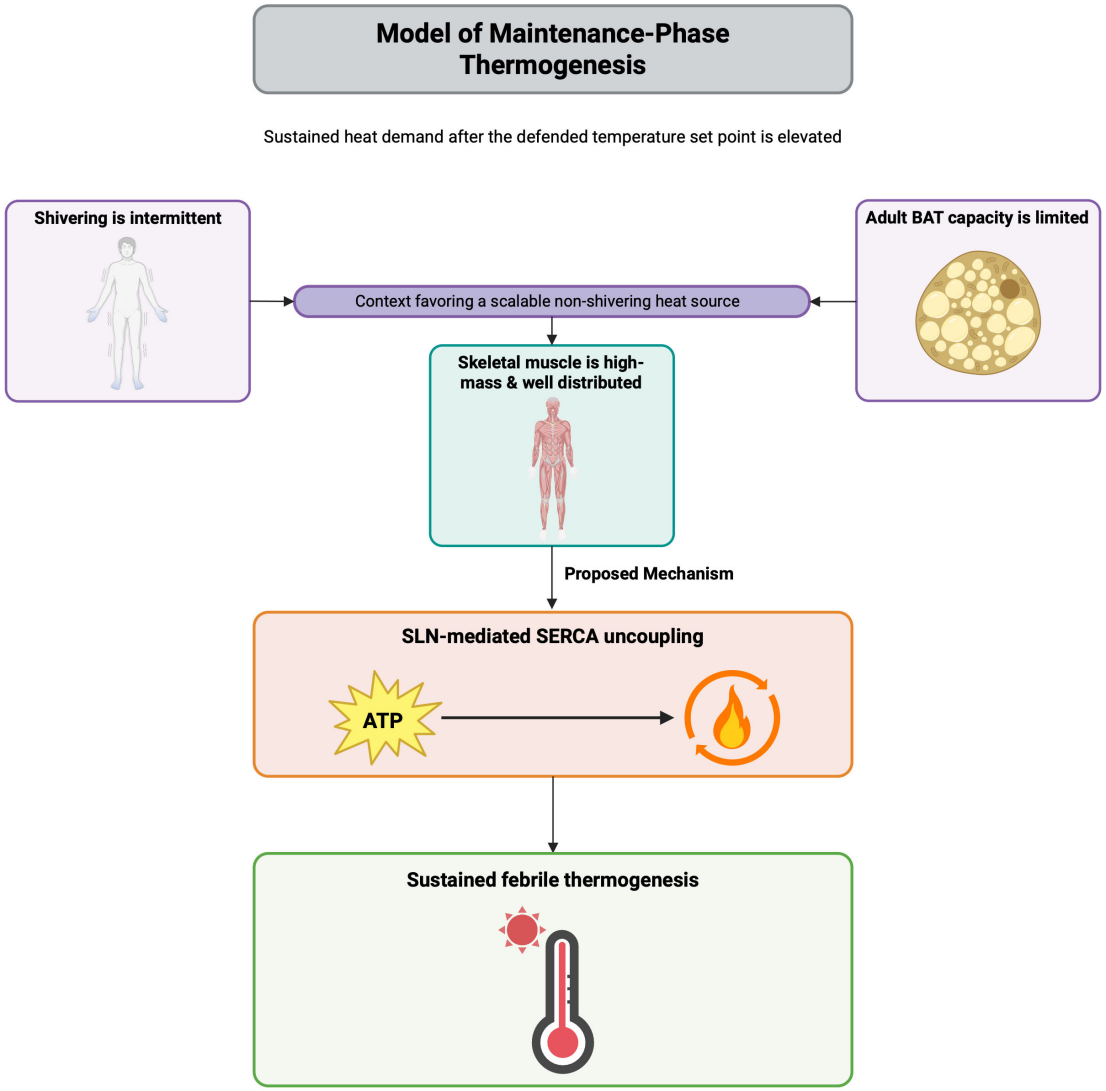
Proposed model of maintenance-phase thermogenesis during fever. Once the defended temperature set point has been elevated, sustained heat production is required to support the febrile plateau. Because shivering is intermittent and adult brown adipose tissue (BAT) capacity is limited, neither mechanism alone may fully account for prolonged febrile heat production. Given its large mass and broad distribution, skeletal muscle is well positioned to provide scalable heat during this phase. In this model, sarcolipin (SLN)-mediated uncoupling of SERCA-dependent Ca^2+^ cycling increases ATP-dependent heat production in resting skeletal muscle, contributing to sustained febrile thermogenesis once overt shivering has subsided. Made using BioRender.

The hypothesis advanced here is that sarcolipin-mediated SERCA uncoupling in skeletal muscle is a scalable contributor to sustaining fever, particularly during the maintenance phase (**Figure 3**). Whereas classical models of febrile thermogenesis emphasize BAT and shivering as the principal heat-producing effectors, the present model proposes that maintenance-phase fever may rely more substantially on skeletal muscle NST mediated by SLN–SERCA uncoupling. In this framework, continuous heat production may arise in part from ATP-dependent Ca^2+^ cycling in resting muscle, with UCP3 serving primarily as permissive metabolic support (**Table 1**).

**Table 1:** Summary of cited evidence supporting the hypothesis that skeletal muscle SLN–SERCA uncoupling contributes to maintenance-phase fever.

Evidence domain	Key references	Main takeaway	Relevance to hypothesis
Central fever control	(Du Bois, 1921; Manthous et al., 1995; Scammell et al., 1996; Exton, 1997; Engblom et al., 2003; Lazarus et al., 2007; Dantzer et al., 2008; Nakamura et al., 2009; Yoshida et al., 2009; Morrison, 2011; Nakamura, 2011; Evans et al., 2015; Walter et al., 2016; Blomqvist and Engblom, 2018; Tan and Knight, 2018; Madden and Morrison, 2019; Morrison and Nakamura, 2019; Wright and Auwaerter, 2020; Pontzer et al., 2021)	Fever is centrally coordinated and increases defended temperature and metabolic demand.	A sustained peripheral heat source is needed once the febrile set point is raised.
BAT in fever	(Astrup et al., 1985; Janssen et al., 2000; Cannon and Nedergaard, 2004; Okamatsu-Ogura et al., 2007; Cypess et al., 2009; Saito et al., 2009; van Marken Lichtenbelt et al., 2009; Virtanen et al., 2009; Yoneshiro et al., 2011; Ouellet et al., 2012; Lee et al., 2013; Blondin et al., 2015; Acosta et al., 2018; Eskilsson et al., 2020; Hachemi and U-Din, 2023; Krysa and Brestoff, 2024; Li et al., 2024)	BAT can contribute to thermogenesis, but adult BAT is limited and UCP1-dependent BAT is not required in several fever models.	BAT alone may not fully explain sustained adult fever.
Shivering	(Martineau and Jacobs, 1988; Castle et al., 1991; Lenhardt et al., 1999; Degroot and Kenney, 2007; Haman et al., 2010; Castellani and Young, 2016; Paal et al., 2016; Walter et al., 2016; Haman and Blondin, 2017; Morrison and Nakamura, 2019)	Shivering is important, especially early, but is intermittent, mechanically expressed, metabolically costly, and constrained by age and host condition.	Supports the idea that maintenance-phase fever may require a non-shivering heat source.
Skeletal muscle energetic capacity	(Zurlo et al., 1990; Janssen et al., 2000; Stewart et al., 2010; Smith et al., 2013; Periasamy et al., 2017; Gamu et al., 2020; Meizoso-Huesca et al., 2022; Singh et al., 2023)	Skeletal muscle is high-mass and a major site of resting ATP turnover; SERCA accounts for a substantial share of resting muscle energy use.	Muscle has the scale to contribute meaningfully to whole-body heat production.
SLN–SERCA uncoupling mechanism	(Smith et al., 2002; Mall et al., 2006; Bal et al., 2012; Bombardier et al., 2013b; Sahoo et al., 2013; Winther et al., 2013; Maurya et al., 2015; Pant et al., 2016; Bal et al., 2018; Maurya et al., 2018)	SLN lowers SERCA coupling efficiency, increasing ATP cost and heat release without force production.	Provides a direct intracellular mechanism for contraction-independent muscle thermogenesis.
Cold-defence and endotherm studies	(Dumonteil et al., 1995; Ouellet et al., 2012; Blondin et al., 2015; Rowland et al., 2015; Bal et al., 2016; Bal et al., 2017; Blondin et al., 2017; Gordon et al., 2019; Nowack et al., 2019; Grigg et al., 2022; Pani et al., 2023; Hutchinson et al., 2026)	Thermogenic load can shift toward muscle, and SERCA-linked heat production appears across endothermic lineages.	Supports muscle NST as a conserved, scalable thermogenic pathway.
UCP3-linked febrile evidence	(Vidal-Puig et al., 2000; Cline et al., 2001; Schrauwen et al., 2003; Brand and Esteves, 2005; MacLellan et al., 2005; Toime and Brand, 2010; Harper, 2016; Riley et al., 2016; Pohl et al., 2019)	UCP3 disruption blunts LPS fever, but the broader literature supports a permissive metabolic role rather than primary heat generation.	Points toward skeletal muscle involvement while supporting SLN–SERCA as the more plausible heat-producing site.
Inflammation-linked support and caveats	(Castle et al., 1991; Kozak et al., 1994; Romanovsky et al., 2005; Pant et al., 2016; Ganeshan and Chawla, 2017; Gordon, 2017; Bal et al., 2021; Costa et al., 2024)	Inflammation is associated with increased SLN expression, but thermal responses vary by model, species, age, and ambient temperature.	Supports the hypothesis but also argues that current evidence remains indirect and context dependent.

Overall, the cited literature supports a model in which skeletal muscle is a plausible maintenance-phase thermoeffector during fever, with SLN-mediated SERCA uncoupling serving as a mechanistically defined candidate pathway. Direct proof of tissue-specific dominance during fever is still lacking.

## 2 Why BAT and shivering may be insufficient to account for sustained fever

BAT and ST remain the two classical explanations for febrile heat production, and both clearly belong within the thermoeffector repertoire. Adult humans do possess metabolically active BAT, and under appropriate stimulation it can contribute measurably to heat production (Cannon and Nedergaard, 2004; Cypess et al., 2009; Saito et al., 2009; van Marken Lichtenbelt et al., 2009; Virtanen et al., 2009; Ouellet et al., 2012; Lee et al., 2013; Hachemi and U-Din, 2023; Krysa and Brestoff, 2024). However, the case for BAT as a complete explanation of sustained fever in adults is limited by scale, variability, and context.

Adult BAT depots are anatomically restricted, highly heterogeneous between individuals, and difficult to quantify precisely, with estimates influenced by imaging approach, cooling protocol, age, and adiposity (Cypess et al., 2009; Saito et al., 2009; van Marken Lichtenbelt et al., 2009; Virtanen et al., 2009; Lee et al., 2013; Hachemi and U-Din, 2023). On average, BAT comprises on the order of less than 0.5% of body weight in adults, against roughly 30–40% for skeletal muscle (Janssen et al., 2000; Hachemi and U-Din, 2023), and whole-body calorimetry during cold exposure indicates that total heat production from skeletal muscle can match or exceed that from BAT largely for this reason (Astrup et al., 1985; Blondin et al., 2015). Activatable BAT also declines with age and adiposity (Cypess et al., 2009; Saito et al., 2009; Yoneshiro et al., 2011), and across individuals its activity correlates only weakly to moderately with the onset and intensity of shivering (Ouellet et al., 2012; Acosta et al., 2018). In the fever literature, the knockout data argue that UCP1-dependent BAT thermogenesis is not required for IL-1β- or LPS-induced fever in mice (Okamatsu-Ogura et al., 2007; Eskilsson et al., 2020), although more recent work indicates that BAT can still participate under selected infectious conditions, including Salmonella infection (Krysa and Brestoff, 2024; Li et al., 2024). Notably, many endotherms defend core temperature without BAT or UCP1 at all, and muscle-based NST arising from Ca^2+^ slippage at SERCA appears evolutionarily ancient and broadly conserved relative to the more recent BAT specialization (Grigg et al., 2022; Grigg, 2025).

Shivering is similarly indispensable to any full account of fever, but it alone does not fully account for febrile heat production, particularly during the maintenance plateau. During the rise in defended temperature, overt muscular activity, rigors, and behavioural cold-defence responses are entirely consistent with an important role for shivering (Walter et al., 2016; Haman and Blondin, 2017; Morrison and Nakamura, 2019). Shivering is mechanically expressed heat production, its intensity varies markedly across muscles and individuals, and its metabolic cost is high (Haman et al., 2010; Haman and Blondin, 2017). At maximal intensity, shivering can raise whole-body metabolic rate to roughly four to five times resting levels (Haman et al., 2010; Paal et al., 2016; Haman and Blondin, 2017), but output of this magnitude cannot be held for long, since sustained shivering drives fatigue, progressive glycogen depletion, and a drift in fuel use and contractile patterning over time (Martineau and Jacobs, 1988; Haman and Blondin, 2017).

While shivering provides a potent source of heat production, it is energetically demanding and increases cardiac work and myocardial oxygen demand (Paal et al., 2016). Furthermore, shivering capacity declines with age because of reductions in muscle mass, motor unit number, and thermoeffector responsiveness (Degroot and Kenney, 2007; Castellani and Young, 2016). These constraints suggest that mechanisms other than shivering may contribute to the maintenance of elevated body temperature during prolonged febrile responses, particularly in older adults (Castle et al., 1991). Even when shivering can be sustained for prolonged periods under cold stress, it remains a variable and behaviourally disruptive strategy (Haman et al., 2010; Haman and Blondin, 2017). This is especially relevant in adult mammals, where the plateau can persist despite the absence of continuous visible shivering. For that reason, shivering is best viewed as a major contributor to febrile heat production, particularly early in the response, but not necessarily as the sole mechanism sustaining the later thermogenic burden.

**Figure 4:**
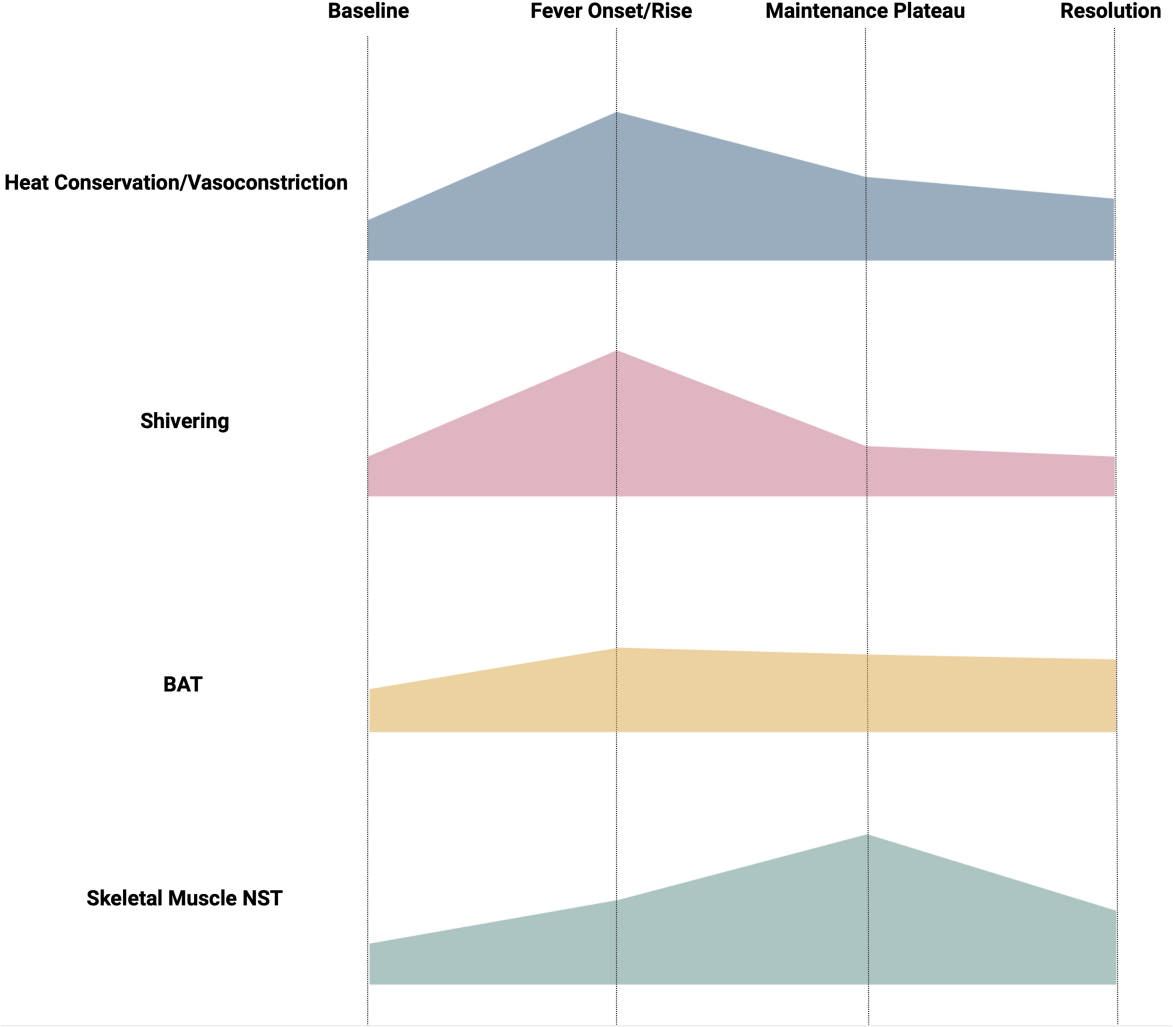
Proposed phase-dependent recruitment of thermoeffectors during fever. This schematic illustrates a qualitative model in which the relative contribution of major thermoeffector pathways shifts across the course of fever. During fever onset and rise, heat conservation/vasoconstriction and shivering are proposed to contribute prominently to the increase in defended body temperature. During the maintenance plateau, sustained heat production may rely relatively more on skeletal muscle non-shivering thermogenesis, while brown adipose tissue (BAT) remains a context-dependent and likely limited contributor in adults. During resolution, the relative contribution of these pathways declines as thermal demand returns toward baseline. This image is conceptual and not intended to indicate exact proportional contributions. Made using BioRender.

A further complication is that much of the mechanistic thermogenesis literature comes from mouse models studied under ambient temperatures that already impose chronic cold stress relative to murine thermoneutrality (Ganeshan and Chawla, 2017; Gordon, 2017). Under those conditions, BAT recruitment and muscle-based thermogenesis are already engaged to defend core temperature, which can blur the distinction between baseline thermoregulatory load and the added energetic demands imposed by inflammatory challenge (Ganeshan and Chawla, 2017; Gordon, 2017). This does not invalidate the mouse fever literature, but it does caution against quantitative extrapolation to adult humans. A thermoeffector that appears dominant in a cold-stressed mouse may carry a different quantitative weight in an adult human under low thermogenic demand, and the relative balance among BAT, shivering, and muscle NST may shift accordingly. Together, these considerations leave room for a contraction-independent heat source in skeletal muscle and support SLN-mediated SERCA uncoupling as a plausible mechanism for sustaining heat production when classical effectors alone do not fully account for the energy balance (**Figure 4**).

## 3 Skeletal muscle as a plausible thermoeffector during fever

At rest, skeletal muscle must continually defend steep sarcoplasmic reticulum (SR) Ca^2+^ gradients, and this maintenance work is costly. SERCA cycles even in quiescent fibres to counter basal Ca^2+^ leak, and integrative analyses place SERCA ATP hydrolysis among the dominant intracellular sinks for resting ATP turnover (Smith et al., 2013; Periasamy et al., 2017; Gamu et al., 2020). In the tightly coupled state, ATP free energy is largely conserved as transport work and expressed as net Ca^2+^ accumulation in the SR. When coupling efficiency is reduced, this relationship changes: ATPase turnover can remain high while net Ca^2+^ sequestration falls, with surplus chemical energy dissipated locally as heat rather than stored in the gradient (Smith et al., 2002). SLN enables this specific shift in pump efficiency without requiring force production. In SR vesicle experiments, SLN depresses Ca^2+^ accumulation disproportionately relative to ATP hydrolysis, the defining signature of uncoupling, thereby increasing the ATP cost per unit Ca^2+^ handled (Smith et al., 2002; **Figure 2**).

This divergence between continued ATP hydrolysis and reduced net Ca^2+^ transport distinguishes SLN-mediated uncoupling from conventional SERCA inhibition. A classical SERCA inhibitor reduces pump turnover, lowering ATP hydrolysis and Ca^2+^ transport in parallel. By contrast, SLN reduces SERCA coupling efficiency: ATP continues to be hydrolysed, but less Ca^2+^ is accumulated per ATP consumed (Smith et al., 2002; Sahoo et al., 2013). As a result, restoring or maintaining SR Ca^2+^ content requires greater ATP expenditure and/or prolonged SERCA activity, with a larger fraction of ATP-derived energy dissipated as heat rather than conserved in the Ca^2+^ gradient. Increased SLN engagement therefore shifts SERCA toward inefficient, ATP-consuming Ca^2+^ cycling, increasing the energetic cost of Ca^2+^ handling and promoting heat production (Smith et al., 2002; Sahoo et al., 2013).

Heat measurements from SERCA-containing membranes support this mechanism by showing that the presence of SLN increases heat output from the Ca^2+^-ATPase itself rather than redistributing work elsewhere in the cell (Mall et al., 2006). Importantly, this regulation is not interchangeable with phospholamban (PLN): SLN–SERCA interactions appear distinct and uniquely favour uncoupling while permitting catalytic cycling, consistent with state-dependent Ca^2+^ slippage within the transport cycle (Sahoo et al., 2013; Winther et al., 2013). In intact muscle, the same directionality holds across levels of analysis: elevating SLN can increase thermogenic capacity and oxygen consumption, whereas SLN deletion lowers the energetic requirement for SR Ca^2+^ transport in resting muscle, indicating that SLN sets coupling efficiency *in vivo* (Bal et al., 2012; Bombardier et al., 2013b; Maurya et al., 2015; Maurya et al., 2018). Together, these findings make it mechanistically credible that muscle can generate sustained heat without shivering, because thermogenesis is driven by intracellular ATPase flux and its regulated inefficiency rather than by overt cross-bridge work.

Direct single-fibre measurements support the existence of this contraction-independent heat source. In resting mammalian muscle, Ca^2+^ leaks through ryanodine receptor 1 (RyR1) into the cytosol, SERCA pumps it back into the SR, and this continuous cycling drives the ATP turnover that generates heat. This capacity appears to be mammal-specific, since leak-driven thermogenesis is markedly greater in mammalian fibres than in amphibians, despite the two sharing the same Ca^2+^-handling machinery (Meizoso-Huesca et al., 2022; Singh et al., 2023). RyR1 leak and SLN act on different points of this cycle, with leak setting the workload imposed on the pump and SLN setting the efficiency with which it converts ATP into transport versus heat. Together, these observations identify resting SERCA cycling as a potential thermogenic pathway through which inflammatory signalling could increase heat production.

Evidence from cold defence studies indicates that facultative thermogenesis is partitioned across effectors and can be reweighted when one component is constrained. In humans, short-term cold acclimation reduces shivering intensity while whole-body heat production is maintained when the cold stimulus is matched (Gordon et al., 2019). Acute cold mapping studies demonstrate that this output reflects the summed response of multiple tissues: BAT is recruited, but skeletal muscle accounts for the dominant share of systemic glucose disposal during cold exposure, even under protocols designed to minimize shivering (Ouellet et al., 2012; Blondin et al., 2015). With longer acclimation, the partitioning can shift again. Four weeks of daily cold exposure increases BAT oxidative capacity, alongside reductions in cold-induced skeletal muscle proton leak and shivering intensity, consistent with a redistribution of the thermogenic load away from muscle uncoupling mechanisms as BAT capacity expands (Blondin et al., 2017). When interscapular BAT is surgically minimized, mice can still cold-adapt and defend core temperature, but they do so at higher energy cost with marked skeletal muscle remodelling of SR and mitochondria and increased SLN expression (Bal et al., 2016). Conversely, under mild-to-severe cold adaptation, loss of SLN biases toward greater BAT recruitment, whereas loss of UCP1 biases toward greater muscle recruitment, indicating bidirectional compensation rather than strict hierarchy (Bal et al., 2017). The requirement for both pathways becomes most apparent in combined loss-of-function models, where UCP1/SLN double knockout mice show profound cold sensitivity during acute cold exposure, consistent with BAT and muscle each providing meaningful, non-trivial capacity to sustain endothermy when the other is limited (Rowland et al., 2015).

A similar partitioning logic has been reported in newborn wild boars, a large mammal lacking BAT, where cold exposure over the first five days of life increased total heat production while shivering intensity decreased; concurrent increases in skeletal-muscle SERCA activity and SERCA1a and SLN expression supported muscle NST as a principal heat source in that setting (Nowack et al., 2019). Within mammals, SLN expression is itself developmentally regulated and substantially higher in the skeletal muscle of larger species than in rodents, and across nutritional and genetic models it tracks whole-body metabolic rate and energy dissipation (Liu et al., 1998; Ukropec et al., 2006; Babu et al., 2007; Bombardier et al., 2013a; Gamu et al., 2015; Pant et al., 2015; Paran et al., 2015; Ballinger et al., 2016; Oliver et al., 2019; Mengeste et al., 2022; Hunstiger et al., 2023). The same reliance on muscle SERCA cycling extends to BAT-lacking birds. Cold exposure and seasonal cold are associated with SERCA- and SLN-related skeletal muscle adaptations in ducklings and adult pigeons, whereas hummingbird torpor data implicate the same axis in the reciprocal suppression of ATP demand and heat production (Dumonteil et al., 1995; Grigg et al., 2022; Pani et al., 2023; Hutchinson et al., 2026). Together, these avian examples indicate that SERCA/SLN regulation can be tuned bidirectionally to raise or lower ATP-dependent heat output (Dumonteil et al., 1995; Grigg et al., 2022; Pani et al., 2023; Hutchinson et al., 2026).

These lines of evidence identify the SLN–SERCA axis as a recurrent site of muscle thermogenesis across species and disparate physiological contexts and show that skeletal muscle has both the scale and the mechanistic capacity to assume a larger share of thermogenic work when required. Together, they make skeletal muscle a tissue in which to look for maintenance-phase febrile heat production.

## 4 Why SLN–SERCA uncoupling is a plausible mechanism for sustaining fever

In the standard knockout tests used to define febrile thermogenesis, UCP1-dependent BAT uncoupling is not necessary for fever expression. IL-1β produces comparable increases in body temperature and activity-corrected metabolic thermogenesis in wild-type and UCP1-deficient mice, indicating that the febrile programme can be executed without UCP1-mediated heat generation in BAT (Okamatsu-Ogura et al., 2007). The same conclusion extends to endotoxin challenge. Following LPS, wild-type and UCP1 knockout mice show similar febrile trajectories, and BAT shows no molecular or substrate-depletion signatures of activation under conditions where a β3-agonist robustly activates BAT, supporting the interpretation that UCP1-dependent BAT thermogenesis is not required for LPS fever in this paradigm (Eskilsson et al., 2020). Krysa and Brestoff summarize recent work showing that fatty acid oxidation is markedly increased in BAT during Salmonella infection-associated fever and argue that BAT can engage a lipid-oxidative programme that may help sustain the febrile response, potentially through thermogenic or metabolic support that is not captured by UCP1-independence alone (Krysa and Brestoff, 2024; Li et al., 2024).

In the same fever paradigms where UCP1 is dispensable, disruption of UCP3 does constrain the response, and the phenotype is most consistent with a skeletal-muscle linked mechanism. In mice lacking UCP3, LPS produces fevers that are both smaller in amplitude and shorter in duration, with attenuated increases in whole-body oxygen consumption, even though BAT structure and UCP1 expression remain intact; selective re-expression of human UCP3 in skeletal muscle partially restores the LPS-evoked thermogenic response (Harper, 2016; Riley et al., 2016). Because UCP3 is expressed predominantly in skeletal muscle, this localizes the principal adjustable uncoupling site to muscle rather than BAT during infection-driven hypermetabolism (Riley et al., 2016; Pohl et al., 2019).

In the original UCP3 knockout characterization, skeletal muscle mitochondria are more tightly coupled and show evidence of higher oxidative stress, and *in vivo* flux analysis indicates greater ATP synthesis relative to TCA cycle flux under fasting conditions, consistent with a role in regulating coupling and stress during high-flux metabolism (Vidal-Puig et al., 2000; Cline et al., 2001). Reviews converge on this interpretation and frame UCP3 as a fatty-acid responsive carrier that can modulate coupling and limit ROS, rather than as a dedicated adaptive thermogenic effector like UCP1 (Brand and Esteves, 2005; Pohl et al., 2019). Several experimental lines support the same permissive function, including: 1) work proposing fatty acid anion export, 2) studies showing increased fatty acid oxidation with lower ROS in muscle cells at physiological UCP3 levels, and 3) direct demonstrations that native UCP3 lowers ROS production in isolated skeletal muscle mitochondria (Schrauwen et al., 2003; MacLellan et al., 2005; Toime and Brand, 2010). In fever terms, these functions would be expected to support sustained oxidative throughput and redox control so that ATP-consuming thermogenic work can be maintained during the plateau.

This interpretation aligns with the muscle effector model, because systemic inflammatory challenges are associated with increased skeletal muscle SLN expression without parallel SERCA expansion (Bal et al., 2021), a pattern consistent with a regulated shift toward less efficient Ca^2+^ cycling and greater heat release (Smith et al., 2002). Rather than implying broad remodelling of the SR Ca^2+^-handling environment, this selective increase suggests that inflammation may act primarily by altering the functional coupling of existing SERCA pumps. Increased SLN engagement could shift SERCA-mediated Ca^2+^ cycling toward greater ATP hydrolysis and heat release within the relevant inflammatory time frame, whereas increased SERCA abundance would be expected to require slower transcriptional and translational remodelling of the SR. Consistent with this, SLN behaves as a state-dependent regulator whose output ranges from limited reductions in SERCA efficiency to marked uncoupling depending on its level and local context (Pant et al., 2016; Bal et al., 2021).

Taken together, the UCP3 data point to skeletal muscle mitochondria as enabling infrastructure for febrile hypermetabolism, whereas SLN-biased SERCA cycling remains a scalable candidate site of heat production in resting fibres (Smith et al., 2002; Mall et al., 2006; Bal et al., 2012; Bombardier et al., 2013b; Sahoo et al., 2013; Winther et al., 2013; Riley et al., 2016; Bal et al., 2018; Bal et al., 2021). On this view, UCP3 would support the oxidative and redox conditions needed to sustain elevated metabolic output, while SLN-SERCA uncoupling would provide a more direct route for converting ATP turnover into heat. This distinction helps reconcile the apparent dispensability of UCP1-dependent BAT thermogenesis with the continued requirement for a muscle-linked thermogenic mechanism during the febrile plateau.

## 5 Discussion

Taken together, these lines of evidence support an integrated model in which immune-to-brain signalling raises defended temperature through PGE_2_ action at EP_3_ receptors in the preoptic region and then recruits a distributed effector network to meet the new thermal demand (Engblom et al., 2003; Lazarus et al., 2007; Nakamura, 2011; Blomqvist and Engblom, 2018; Tan and Knight, 2018; **Figure 1**). Preoptic command is relayed through parallel autonomic and somatic pathways that coordinate heat conservation and heat production, including cutaneous vasoconstriction, recruitment of shivering circuitry, and sympathetic activation of BAT when that tissue is available and responsive (Scammell et al., 1996; Nakamura et al., 2009; Yoshida et al., 2009; Morrison, 2011; Nakamura, 2011; Tan and Knight, 2018; Madden and Morrison, 2019). This architecture explains why fever expression is robust across contexts even when individual effectors are constrained.

Stress-induced (psychogenic) hyperthermia is another condition in which the central nervous system actively elevates and defends body temperature (Nakamura, 2015; Oka, 2015). Similar to fever, this response is centrally mediated and can occur independently of UCP1-dependent thermogenesis (Eskilsson et al., 2020). Exposure to predator odour, a commonly used psychological stressor, reduces SERCA Ca^2+^-transport efficiency in skeletal muscle and contributes to the resulting increase in heat production (Gorrell et al., 2020; Heemstra et al., 2022; Gorrell et al., 2025). The acute nature of stress hyperthermia demonstrates that skeletal muscle SERCA thermogenesis can be rapidly recruited to increase heat production, although these studies do not address its contribution to the prolonged maintenance of elevated body temperature during fever. Whether the same underlying mechanisms are engaged in both conditions remains unknown.

The distributed-effector model proposed here therefore requires a temporal distinction between fever initiation and fever maintenance (**Figure 4**). Shivering and BAT thermogenesis can be recruited rapidly through central thermoregulatory command to initiate fever (Scammell et al., 1996; Nakamura et al., 2009; Yoshida et al., 2009; Morrison, 2011; Nakamura, 2011; Tan and Knight, 2018; Madden and Morrison, 2019), whereas an inflammation-associated increase in SLN abundance would more likely act as a slower and more persistent thermoregulatory source. This temporal mismatch argues that SLN-SERCA uncoupling is a likely maintenance-phase contributor. During this maintenance plateau, a substantial component of continuous heat production may arise from skeletal muscle NST, expressed as increased ATP turnover in resting fibres via SLN-biased SERCA Ca^2+^ cycling, with minimal mechanical output (**Figure 3**). Within this framework, UCP3 is positioned as permissive support rather than primary heat source. Its role is proposed to be preservation of sustained oxidative flux and redox tolerance in skeletal muscle mitochondria during prolonged immune challenge (Vidal-Puig et al., 2000; Cline et al., 2001; Schrauwen et al., 2003; Brand and Esteves, 2005; MacLellan et al., 2005; Toime and Brand, 2010; Harper, 2016; Riley et al., 2016; Pohl et al., 2019).

The strength of this model is that it brings together several observations that are otherwise difficult to reconcile in a single framework (**Table 1**). UCP1-independent fever argues against a simple BAT-centric account in adult animals (Okamatsu-Ogura et al., 2007; Eskilsson et al., 2020). The UCP3 phenotype points toward skeletal muscle, but the broader UCP3 literature does not support the idea that UCP3 itself is the major adaptive heat source (Vidal-Puig et al., 2000; Cline et al., 2001; Schrauwen et al., 2003; Brand and Esteves, 2005; MacLellan et al., 2005; Toime and Brand, 2010; Harper, 2016; Riley et al., 2016; Pohl et al., 2019). At the same time, SLN–SERCA uncoupling provides a well-defined intracellular mechanism through which ATP hydrolysis can be converted into heat without overt contraction (Smith et al., 2002; Mall et al., 2006; Bal et al., 2012; Bombardier et al., 2013b; Sahoo et al., 2013). Finally, inflammatory increases in SLN expression without parallel changes in SERCA abundance are consistent with regulatory tuning of existing machinery (Bal et al., 2021). Considered together, these points make skeletal muscle SERCA uncoupling a coherent candidate mechanism for sustaining fever.

At the same time, the evidence remains indirect. Much of the mechanistic support for SLN-mediated thermogenesis comes from resting muscle, cold adaptation, membrane preparations, and genetic models not designed specifically to resolve tissue-level heat production during fever itself. Likewise, the inference from increased SLN expression to febrile heat output is plausible but not yet mechanistically established. An additional point is that the contribution of skeletal muscle thermogenesis during fever probably shifts with context (**Figure 4**). Fever is a variable physiological state rather than a single fixed phenotype, and the same inflammatory challenge can produce different thermal outcomes depending on species, ambient temperature, dose, and time after challenge (Kozak et al., 1994; Romanovsky et al., 2005; Costa et al., 2024). This matters in translational work because mice are often studied below thermoneutrality and rely on thermoeffector strategies that do not map cleanly onto adult humans (Ganeshan and Chawla, 2017). Age likely matters as well, since older individuals often have lower baseline temperatures and a blunted febrile response to infection, which could alter both the magnitude of the metabolic response and the apparent partitioning of heat production (Castle et al., 1991). Infection model and inflammatory severity should also be expected to matter. Mild or moderate inflammatory challenges may favour a sustained febrile programme, whereas severe systemic inflammation can shift the thermal phenotype toward hypothermia, especially under cooler ambient conditions (Romanovsky et al., 2005; Costa et al., 2024). Viewed this way, SLN–SERCA uncoupling is most likely to matter in adult maintenance-phase fever, when resting muscle can sustain elevated ATP-dependent heat production (Romanovsky et al., 2005; Ganeshan and Chawla, 2017; Costa et al., 2024).

A further strength of the hypothesis is that it is experimentally testable within the framework of current knowledge. If SLN-mediated SERCA uncoupling contributes materially to maintenance-phase fever, then perturbing SLN specifically in skeletal muscle should blunt maintenance-phase increases in VO_2_ or heat production more than it blunts the initial centrally driven rise in defended temperature. Likewise, this reduction should occur without a proportional fall in overt electromyographic shivering, helping distinguish contraction-independent heat production from mechanical thermogenesis. If UCP3 is mainly permissive, then altering UCP3 should modify the ability to sustain oxidative throughput but not necessarily identify the primary intracellular site at which heat is generated. More direct approaches could combine indirect calorimetry, core temperature telemetry, electromyography, isotope-based flux analysis, and tissue-specific genetic or pharmacologic perturbation to quantify skeletal muscle heat production during fever and separate SERCA-dependent Ca^2+^ cycling from shivering. In larger mammals and translational settings, pairing calorimetry with muscle activity measures and imaging-based assessments of BAT activation would be especially important.

The hypothesis also raises several testable mechanistic questions. Does inflammatory signalling increase SLN expression sufficiently and quickly enough to influence fever maintenance within the relevant time window? Is the effect generalized across multiple muscle groups, or concentrated in specific fibre types or anatomical regions? Does the contribution of SLN–SERCA uncoupling increase when BAT capacity is constrained, or when shivering is minimized by behavioural or environmental context? Furthermore, does the proposed mechanism account mainly for maintenance of the plateau, or does it also contribute to the later phases of fever resolution? These questions would help determine whether skeletal muscle SERCA uncoupling should be regarded as a major physiological effector, a context-dependent amplifier, or one component within a broader distributed thermogenic network.

In summary, the available evidence supports a model in which sarcolipin-mediated SERCA uncoupling in skeletal muscle is a scalable candidate mechanism for sustaining fever, particularly during the maintenance phase when shivering is intermittent and adult BAT capacity is limited (**Figure 3**). However, this framework does not yet establish SLN–SERCA uncoupling as the directly demonstrated dominant heat source in fever. Definitive resolution will require direct quantification of tissue-specific heat production and ATP turnover during fever, coupled with perturbation strategies that separate contraction-dependent thermogenesis from SLN–SERCA-driven Ca^2+^ cycling. Determining whether skeletal muscle SERCA uncoupling functions as a major febrile effector would refine current models of thermoregulation and identify a mechanistically distinct pathway through which inflammation can raise metabolic rate and defended body temperature.

## Data Availability

The original contributions presented in the study are included in the article/supplementary material. Further inquiries can be directed to the corresponding author.
